# The effect of posterior tibial slope on anteroposterior stability in posterior cruciate retaining total knee arthroplasty

**DOI:** 10.1186/s12891-023-06507-6

**Published:** 2023-05-16

**Authors:** Mehmet Ersin, Mehmet Demirel, Melih Civan, Mehmet Ekinci, Turgut Akgül, Cengiz Şen

**Affiliations:** 1 Istanbul Haseki Training and Research Hospital, Fatih/İstanbul, Türkiye; 2 Istanbul Faculty of Medicine, İstanbul University, Fatih/İstanbul, Türkiye; 3 Basaksehir Cam and Sakura City Hospital, University of Health Sciences, Istanbul, Türkiye

**Keywords:** Posterior tibial slope (PTS), Total knee arthroplasty, KT-1000 arthrometer, Anteroposterior laxity

## Abstract

**Background:**

It has been suggested that the posterior tibial slope (PTS) plays an important role in increasing the anteroposterior stability following total knee arthroplasty. Although the relationship between the PTS and the flexion range has been investigated many times, studies on the relationship between PTS and anterior-posterior stability are limited. The primary aim of this study was to investigate the relationship and effects of PTS on anteroposterior stability in posterior cruciate retainer total knee arthroplasty.

**Methods:**

154 primary TKAs were identified retrospectively to analyze the any association between PTS and anteroposterior laxity following posterior cruciate-retaining total knee arthroplasty in the overall study populations. Anteroposterior displacement was measured at the final follow-up based on the following two procedures: KT-1000 arthrometer and sagittal drawer radiographic images. In addition, the relationship between PTS and functional scores-ROM was examined.

**Results:**

There was no correlation between patients’ posterior tibial slope and postoperative VAS (r: -0.060, p:0.544), WOMAC (r:0.037, p:0.709), KSS (r: -0.073, p:0.455). In addition, there was no significant correlation between postoperative knee ROM and postoperative PTS (r:0.159, p:0.106). Moreover, no correlation was found between KT-1000 arthrometer and 20 degrees AP translation with PTS. There was a negative correlation between PTS and 70 degrees AP translation (r: -0.281, p:0.008).

**Conclusions:**

This study aimed to clarify the association between instability and AP laxity in flexion of implanted knees, and to determine what degree of AP laxity results of instability. A fundamental finding of this study was that; the optimum TS angle to increase anterior-posterior stability after total knee arthroplasty is between ≥ 4 to < 6 degrees, we also proved that there is no relationship between stability and patient satisfaction.

## Introduction

One of the most fundamental goals of total knee arthroplasty is to ameliorate the functional range of motion without causing instability [1]. Due to its significant impact on postoperative knee function and prosthesis stability, anteroposterior stability (displacement) is considered a pivotal factors that affect the clinical success of TKA [2].

Despite controversial findings in the literature, PCL is suggested to play an essential role in promoting anteroposterior stability following total knee arthroplasty. When this ligament is retained, it is considered to support knee stability by limiting posterior translation [3]. Accordingly, many biomechanical and clinical studies have focused on the role of PCL in maintaining anteroposterior stability following TKA, and the effects of prosthetic designs retaining or sacrificing this ligament on anteroposterior displacement have been assessed by various methods to date [4–6].

The posterior tibial slope (PTS) is proposed to be a key factor in promoting proper knee biomechanics and especially the stability of PCL. Studies indicate that PTS is positively associated with the flexion range and prevents extreme tension on the PCL during knee flexion [3, 7]. Thus, we assumed that changes in PTS following PCL-retaining TKA would have a significant impact on anteroposterior stability. However, according to our review of the literature, little is known about the relationship between PTS and anteroposterior stability.

The primary aim of this study was to investigate the association and effects of PTS on anteroposterior stability in posterior cruciate-retaining total knee arthroplasty.

## Materials and methods

After obtaining approval from the institutional review board, patients who underwent TKA with a diagnosis of OA between December 2013 and February 2015 were retrospectively reviewed. A total of 161 consecutive primary TKAs were identified retrospectively. After obtaining informed consent from the eligible patients, the current study was carried out in two phases: (1) to analyze whether there is any association between PTS and anteroposterior laxity following posterior cruciate-retaining total knee arthroplasty in the overall study populations and (2) to determine the effect of PTS on anteroposterior laxity based on the subgroup analyses.

**Fig. 1:**
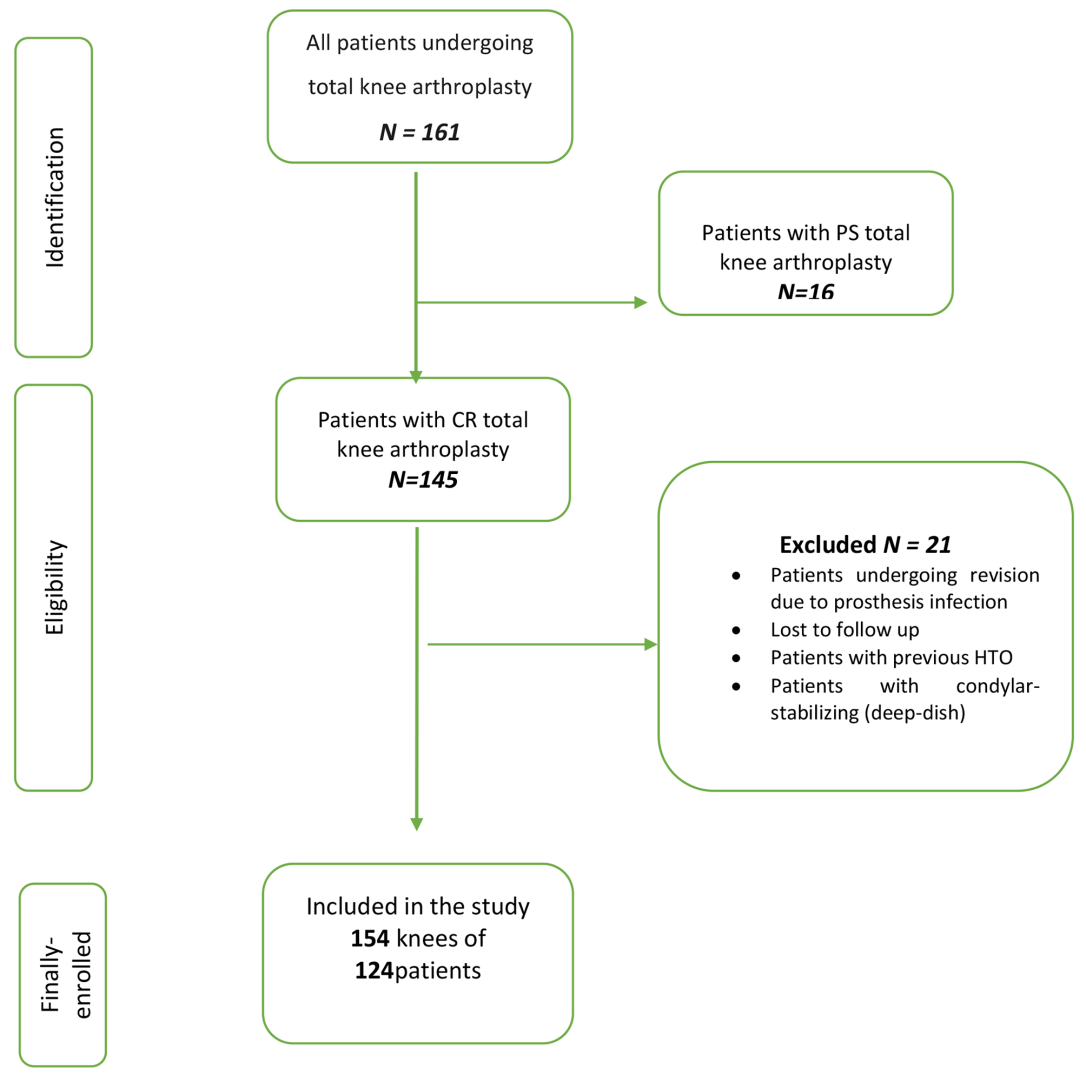
Flow chart of the study

Figure 1 demonstrates the flow chart of the study participants.

### Patients

The mean age was 63.39 ± 7.52 years (range, 50.1–78.5), and the mean follow-up was 33.59 ± 7.24 months (range, 24– 48).

### Outcome measures

#### Assessment of PTS and anteroposterior laxity of the knee joint

The postoperative PTS was measured based on the proximal tibial anatomical axis described by Utzschneider et al. [8], using the software package AUTOCAD 2017 on each patient’s final follow-up lateral radiograph images [9, 10].

Anteroposterior displacement was measured at the final follow-up based on the following two procedures: (1) KT-1000 knee joint arthrometer (MEDmetric Corp., San Diego, CA) and (2) sagittal drawer radiographic images.

KT-1000 arthrometer was performed by an experienced orthopedic surgeon with clinical experience using the device. The amount of tibial displacement was measured at 30° flexion when an anterior tibial translational force of 67 N, 89 N, and a posterior force of 134 N were applied to the lower leg [11]. Measurements were made three times consecutively, and the average value of 3 measurements was used.

**Fig. 2:**
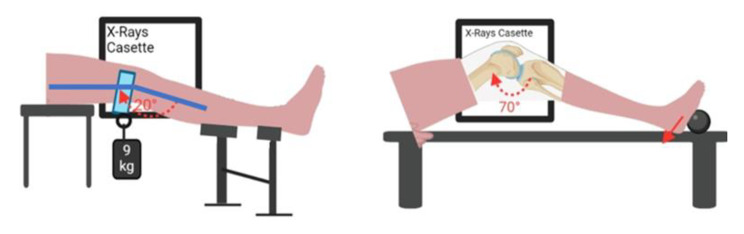
A-Position on the special device for measurement of anterior translation of the knee (I, calf support to examine the knee at 20°; II, height-adjustable support for the foot)

Sagittal radiographic drawer images were obtained according to the passive anterior drawer and active posterior drawer testing procedures (Fig. 2) [12]. This procedure was performed in a special setup, as indicated in the literature (Fig. 2) [12]. Anterior and posterior translations of the tibia with respect to the femur were then measured on sagittal radiographs using the software package AUTOCAD 2017 (Sausalito, CA, USA).

#### Clinical outcome measures

In addition to the knee flexion range of motion (ROM), the Knee Society Score (KSS), Western Ontario and McMaster Universities Osteoarthritis Index (WOMAC) score [13], and visual analog scale (VAS) were undertaken for each patient at the final follow-up by the same orthopedic surgeon. ROM was measured using a universal standard goniometer with the patient in the supine position as described previously [14]. VAS was used to evaluate changes in pain intensity. The VAS score utilized in the present study is a modified and simplified measure in which pain intensity during daily activity is rated on a scale of 0–10, where 0 indicates no pain and 10 indicates the worst pain.

The primary outcome of this study was to assess whether there is any correlation between postoperative PTS and anteroposterior displacement. The secondary outcome was to assess the correlation between postoperative PTS and clinical outcome measures (Table 1).

**Table 1:** Correlation analyses between PTS and clinical outcomes in the overall study population

	Flexion ROM	KSS	WOMAC Score	VAS
Tibial Slope Angles r	0.159	-0.073	0.037	-0.060
P*	0.106	0.455	0.709	0.544

**ROM** = range of motion; **KSS** = Knee Society Score; **WOMAC** = Western Ontario and McMaster Universities Osteoarthritis index; **VAS** = Visual Analogue Scale

*The correlation is significant at the level of *P* < .05.

### Surgical technique

All operations were performed by senior orthopedic surgeons, using a midline skin incision followed by a standard medial parapatellar approach. An appropriately sized pneumatic thigh tourniquet was applied to all patients. All patients underwent the GENESIS II™ cemented, posterior-cruciate-retaining, fixed-bearing total knee endoprosthesis with ultra-high molecular weight polyethylene (Smith & Nephew, Memphis, TN, USA). Cutting blocks were aligned using an extramedullary guide in the tibia and an intramedullary guide in the femur. As with many brands, there was a 3° of posterior slope inherent to the polyethylene.

In all patients, no severe bone deficiency that required augmentation or bone grafting existed, and PCL was intact in terms of appearance and tension during the operation. Additionally, soft tissue balancing was arranged to obtain valgus and varus stability. Based on our institutional experience, the patella was not routinely replaced, and only marginal osteophytes were resected.

### Statistical analysis

Statistical software package SPSS 20.0 (IBM Corp, 2011, Armonk, New York) was used for analysis. Statistical significance was set at P < .05. The test for normality of the variables was done by Shapiro–Wilk Test. Descriptive data are given as frequencies, percentages, means, and standard deviations or medians and ranges (minimum and maximum). Comparisons were undertaken using paired sample t-test for normally distributed continuous variables and Pearson’s chi-square test, and Fisher’s exact test for categorical variables. Comparisons among multiple groups were conducted using the one-way ANOVA test for normally distributed continuous variables and a one-sided analysis of covariance (ANCOVA) for WOMAC to adjust for gender as a potential covariate. Correlations were analyzed using a Pearson analysis for parametric data.

To assess the association between PTS and anteroposterior laxity at the final follow-up, statistical analyses were first performed in the overall study populations (Table 2) then stratified with KT-1000 (< 3 mm and ≥ 3 mm) considered pathological in addition to the anterior-posterior displacements according to PTS and radiographic analysis.

**Table 2:** Descriptive statistics of the tibial slope and anteroposterior displacement measurements at the final follow-up

	*Min* – *Max*	Mean ± SD
**Tibial slope (°)**	-2.5–13.5°	4.63 ± 2.99
**Anterior displacements as per KT-1000 (mm)**		
**67 N** **89 N** **134 N**	0.5–9.5 1–10.5 2–12	4.39 ± 2.18 4.77 ± 2.21 5.32 ± 2.41
**Posterior displacements as per KT-100 (mm)**		
**67 N** **89 N** **134 N**	0–4 0–6 0–7	0.85 ± 0.75 1.42 ± 0.91 1.64 ± 1.12
**PAD measurements at 20° of flexion** **APD measurements at 70° of flexion**	0–22.9 0–19.7	6.77 ± 5.46 3.63 ± 3.24

PAD = passive anterior displacement; APD = active posterior displacement

## Results

Based on the eligibility criteria 124 patients (154 knees) were included in the study and invited to a final follow-up examination (Fig. 1).

### Outcomes of the overall study population

Post-op KSS, WOMAC, VAS, and slope values of the patients are detailed in Table 3. Tibial slope and postop VAS (r: -0.060, p:0.544), WOMAC (r:0.037, p:0.709), KSS (r: -0.073, p:0.455) values of the patients were evaluated with Pearson correlation test. We revealed that there is no correlation between the tibial slope and the KSS, WOMAC, and VAS scores. No significant correlations were found between postop knee ROM and postoperative slope values (r:0.159, p:0.106) (Table 1).

**Table 3:** Descriptive statistics of the clinical outcomes at the final follow-up

Flexion ROM (°)	*Min* – *Max*	70–135°
	*Mean ± SD*	108.45 ± 14.94
**KSS**	*Min* – *Max* *Mean ± SD*	(47–100) 87 ± 10
**WOMAC score**	*Min* – *Max*	34–100
	*Mean ± SD*	83 ± 13
**VAS**	*Min* – *Max*	0–5
	*Mean ± SD*	1 ± 1

ROM = range of motion; KSS = Knee Society Score; WOMAC = Western Ontario and McMaster Universities Osteoarthritis index; VAS = Visual Analogue Scale

### Association between PTS and anteroposterior laxity

In the measurements evaluated with the KT-1000 knee joint arthrometer, the mean anterior translation values at 67 N, 89 N, and 134 N were 4.39 ± 2.18 mm, 4.77 ± 2.21 mm, and 5.32 ± 2.41 mm, respectively (Table 2). It was shown that there was no correlation between the KT-1000 knee joint arthrometer and the tibial slope (67 N, 89 N, and 134 N, r:0.012/p:0.906, r:0.01/p:0.993 and r:0.018/0.886, respectively). Mean posterior translation values (67 N, 89 N, and 134 N) were 0.85 ± 0.75 mm, 1.42 ± 0.91 mm, and 1.64 ± 1.12 mm, respectively, and there was no correlation with tibial slope (r: -0.058). /p:0.556, r: -0.024/p:0.806 and r: -0.017/0.738, respectively).

Measurements performed on sagittal drawer x-rays, the mean (± SD) anteroposterior translation value of the patients at 20 degrees of flexion (PAD: passive anterior displacement) were 6.77 ± 5.46 (0 to 22.9) mm, and the mean anteroposterior translation value at 70 degrees of flexion (APD: active posterior displacement) were 3.63 ± 3.24 (0 to 19.7) mm. The relationship between the tibial slope and translation was examined. No correlation was found between 20 degrees AP translation and tibial slope (r:0.200, p:0.065). There was a negative correlation between the tibial slope and 70 degrees AP translation (r: -0.281, p:0.008). While the tibial slope does not affect translation at 20 degrees of flexion, increasing the tibial slope at 70 degrees reduces translation. However, it is obvious that this correlation is weak (r: -0.281), and therefore the effect of the tibial slope on AP translation is not very important even in 70 degrees of flexion.

### The effects of PTS on anteroposterior laxity based on the subgroup analyses

As it is widely accepted in the literature, we accepted 3 mm as the pathological limit of the KT-1000 knee joint arthrometer [11]. We compared non-pathological (< 3 mm) and pathological (≥ 3 mm) values (at 67 N, 89 N, and 134 N) and revealed that there was no significant relationship between tibial slope and KT-1000 knee joint arthrometer values (0.845, 0.884 and 0.823, respectively).

**Fig. 3:**
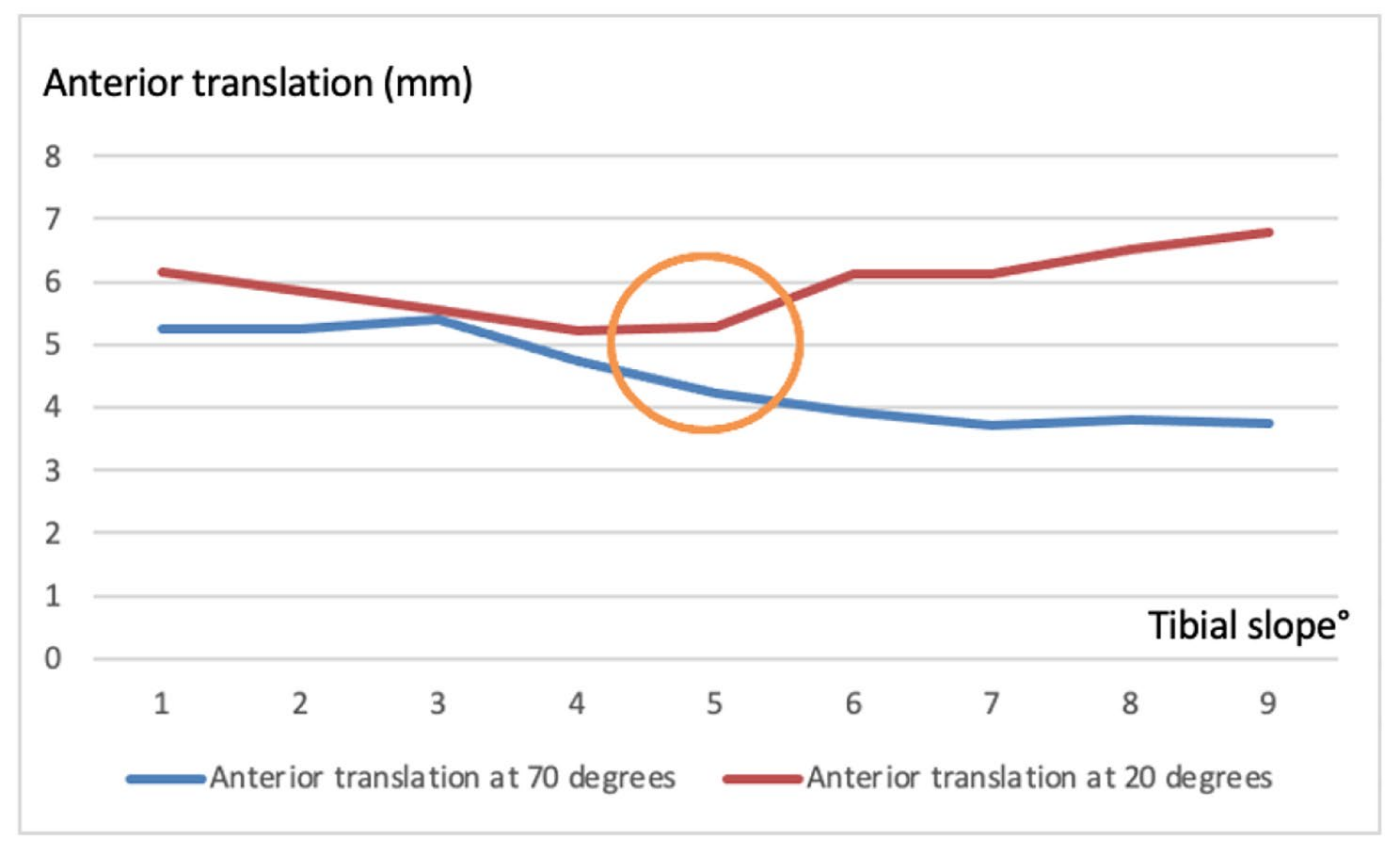
Relationship of AP translation with PTS

In subgroup analyzes, on sagittal drawer x-rays, we observed that a tibial slop ≥ 6 degrees reduces anterior translation at 70 degrees. Meanwhile, we noticed that a tibial slope ≥ 4 to < 6 degrees at 20 degrees reduces anterior translation. Both 20 degrees and 70 degrees minimum AP translation were observed to be at 4–6 degrees tibial slope (Fig. 3). After determining the optimum PTS angle, we divided them into three groups (group1 < 4 degrees, group2 ≥ 4–6 degrees, and group3 ≥ 6 degrees) and analyzed them again. We found that there were significant differences between Group 2, which we thought to be the ideal angle, and group 1 and group 3 comparison groups (anterior translation at 20 degrees and anterior translation at 70 degrees, p:0.048, p:0.001, respectively).

## Discussion

Many factors, such as prosthesis design, soft tissue, the tension of the surrounding soft tissue, tibial slope, and PCL, play an important role in TKA stability. In sagittal balance, several (clinical) studies have shown that the posterior tibial slope (PTS) has an important role in the biomechanics of the knee joint and the stability of the posterior cruciate ligament. It is, therefore, suggested that PTS makes a significant impact on the clinical and functional results of total knee arthroplasty (TKA) [15].

There are limited studies in the literature on the effect of PTS on functional knee scores. In our study, we also examined the relationship between PTS and postop WOMAC and KSS scores and revealed that there was no relationship between them (p:0.709, p:0.455, respectively). Similarly, in the literature, Shi et al. reported that there was no relationship between PTS and postop WOMAC and KSS scores. We also examined the postop VAS scores and the pre-and postoperative changes of all scores, unlike previous studies. We showed that increasing or decreasing the PTS did not lead to any improvement in functional scores.

It is generally believed that knees with a greater tibial slope have greater knee flexion [16, 17]. Although many articles in the literature found a correlation between the increase in PTS and maximal flexion, we could not find a relationship between PTS and maximal flexion in our study. Since the available literature studies are usually cadaveric or finite element studies, clinical results have not been investigated in all aspects. Furthermore, PTS has been reported to affect posterior tibiofemoral translation [3]. However, we could not detect such a relationship.

Anteroposterior instability does not directly indicate posterior cruciate ligament insufficiency. However, since the posterior cruciate ligament is the primary constraint in anterior-posterior instability when the knee is flexed, the increase in anterior-posterior laxity at 75 degrees of flexion may reflect posterior cruciate ligament insufficiency [11]. Anteroposterior stability is evaluated by various methods; in the literature, the KT-1000 knee joint arthrometer is one of the methods evaluating anterior-posterior stability after posterior cruciate ligament-retaining total knee arthroplasty [11, 18, 19].

The relationship between instability, which was evaluated with the KT-1000, and knee ROM has been investigated many times in the literature [18, 19]. In our study, we examined the relationship between KT-1000 and PTS; however, we found no relationship between them. Then, we performed subgroup analyzes as in the literature and considered a total of 10 mm above the anterior and posterior translation measured by KT-1000 as pathological. We performed subgroup analyzes as in the literature, and we considered the anterior ≥ 3 mm and posterior < 2 mm translation measured with the KT-1000 as pathological. Values with a total anterior and posterior translation > 10 mm were also considered pathological [18]. Finally, we did not find a relationship between PTS and KT-1000 in subgroup analysis.

Measurements performed on sagittal drawer x-rays, the mean (± SD) anteroposterior translation value of the patients at 20 degrees of flexion (PAD) were 6.77 ± 5.46 (0 to 22.9) mm, and the mean anteroposterior translation value at 70 degrees of flexion (APD) were 3.63 ± 3.24 (0 to 19.7) mm. The relationship between the tibial slope and translation was examined. No correlation was found between 20 degrees AP translation and tibial slope (r:0.200, p:0.065). There was a negative correlation between the tibial slope and 70 degrees AP translation (r: -0.281, p:0.008). While tibial slope does not affect translation at 20 degrees of flexion, increasing tibial slope at 70 degrees reduces translation. However, it is obvious that this correlation is weak (r: -0.281) and therefore the effect of the tibial slope on AP translation is not very important even in 70 degrees of flexion.

We evaluated the relationship between 20 degrees of flexion (PAD) values ​​and 70 degrees of flexion (APD) values ​​with PTS. There was no correlation between PAD and PTS, but we noticed a negative correlation between APD values ​​and PTS (p:0.008). In other words, we showed that anterior-posterior translation decreases as PTS increases at 70 degrees of flexion. Although this was a significant value, it was a weak correlation (r: -0.281) (Fig. 3).

The current study has some limitations. PCL tension is affected by many factors, such as, PTS, flexion gap, thickness of bone resection for proximal tibia and posterior femur, and choice of implant size/position. PTS is only one of these many factors, but the flexion gap has been adjusted intra-operatively by senior surgeons. Evaluation using the KT-1000 arthrometer is static. The return of the femoral component, which is another important role of the posterior cruciate ligament, was not evaluated. Another limitation is that it is not compared with posterior stabilized total knee arthroplasty [11]. Despite all these limitations, we evaluated the relationship between PTS and anterior-posterior translation using more than one method. In addition, this study is one of the rare studies that examines the effects of PTS in such detail.

This study aimed to clarify the association between instability and AP laxity in flexion of implanted knees, and to determine what degree of AP laxity results of instability. If this were clarified, the clinical relevance would be that the target area of AP laxity in TKA is determined. A fundamental finding of this study was that; the optimum PTS angle to increase anterior-posterior stability after total knee arthroplasty is between ≥ 4 to < 6 degrees, we also proved that there is no relationship between stability and patient satisfaction.

## Data Availability

The datasets used and/or analyzed during the current study are available from the corresponding author on reasonable request.
